# Distinguishing among evolutionary and ecological processes shaping microbiome dynamics

**DOI:** 10.1093/ismejo/wraf107

**Published:** 2025-05-23

**Authors:** Tiffany N Batarseh, Britt Koskella

**Affiliations:** Department of Integrative Biology, University of California Berkeley, Berkeley, CA 94710, United States; San Francisco Chan Zuckerberg Biohub, San Francisco, CA 94158, United States; Department of Integrative Biology, University of California Berkeley, Berkeley, CA 94710, United States; San Francisco Chan Zuckerberg Biohub, San Francisco, CA 94158, United States

**Keywords:** evolution, ecology, microbiomes, bacteria, genomes, metagenomics, microbial function

## Abstract

Evolution is defined as the change in allele frequency over time as a result of either neutral processes, such as genetic drift, or as an adaptive process in response to selection. In contrast, ecological dynamics describe changes in population densities, species distributions, species interactions, and/or relative abundances within communities, all of which can also be the result of either stochastic or deterministic processes. Although the distinction between these patterns has long held for plants and animals, microbial community dynamics can blur the line between ecological and evolutionary processes, especially as they can occur on very similar timescales. Despite the importance of differentiating changes occurring within a population or strain from those occurring among populations, many common methodologies used to study microbiomes are not able to differentiate among them. In this review, we summarize the forces known to generate genetic diversity in bacterial genomes and describe the approaches used to study bacterial evolution from simple to more complex systems. We then explore how current methodologies and conceptual understanding can be applied to both understand and differentiate between the ecological and evolutionary processes in microbial communities. By highlighting lessons from longitudinal microbiome studies and experimental evolution, we explore the unique opportunities afforded by newer sequencing approaches and better sequencing resolution. Throughout, we identify the unique and outstanding challenges in studying these processes in microbiome systems and emphasize the great benefits in doing so to move forward our ability to better predict and manipulate microbiomes.

## Introduction

Microbial populations and communities are highly dynamic over space and time. Studying the drivers of these dynamics is key to predicting, understanding, and/or manipulating microbiomes. These are critical goals given the importance of microbiomes to the function of many systems including plants and animals, where these complex microbial communities can impact host reproduction [1], nutrient acquisition [2], pathogen susceptibility, [3, 4] and much more [5]. As the microbiome can shape host phenotype and reshape the distribution of host genotypes across environments, it is imperative that we understand the processes governing its evolution (including the fate of intraspecific variants and spread of new alleles) and ecology (including changes in community composition and microbial interactions). Outside of the eukaryotic host, bacteria are ubiquitous organisms responsible for proper ecosystem functioning and nutrient cycling, and thus the study of ecological and evolutionary processes of environmental microbiomes also holds great value. In all cases, the responsiveness of microbial populations and communities to local biotic and abiotic selection (at ecological and evolutionary scales) can mean flexibility in function, resilience to changing environments, and knock-on effects on the evolution and ecology of plant and animal communities that rely upon them.

Across both observational studies in which taxonomic composition of microbiomes is described (e.g. [6–9]) and experimental studies in which microbiomes and/or selection are directly manipulated (e.g. [10, 11]), it has proven challenging to differentiate ecological changes in species presence and abundance from evolutionary changes resulting from either strain replacement by migration or de novo mutation. Our understanding of microbiome ecology comes primarily from observational studies in which microbial diversity (i.e. which taxa are present and how abundant they are relative to one another) is measured across either space (e.g. [6, 8]) or time (e.g. [6]), often guided by hypotheses regarding the selection pressures that might vary across these samples. Such studies have underscored the importance of dispersal, selection, and within-microbiome interactions in shaping community composition, but often fall short of pinpointing mechanisms underlying such change. In contrast, our current understanding of bacterial evolution comes primarily from experiments conducted most typically using single species in controlled laboratory conditions [12, 13]. Experimental evolution of monocultures has given remarkable insight to the tempo [14], mode [15], and molecular underpinnings of adaptive evolution [16, 17], but, as developed below, these findings likely may not directly translate to complex environments and larger microbial communities.

Communities provide novel selection pressures that, until recently, have been difficult or impossible to capture in either traditional evolution experiments or microbiome sequencing efforts. Most obviously may be the biotic pressures imposed between and among the microbes themselves [18, 19] and even across biological kingdoms, such as the predation of bacteria by protists. Additionally in complex communities, organisms are known to modify their environment which can reshape subsequent selection pressures felt by other community members. This process, referred to as niche construction [20], has important implications for evolutionary processes and ecosystem ecology but is hard to study *in situ*. Host-associated microbial taxa face the additional selection pressures that come from host life history, such as birth mode [21], host dietary differences [11], and even domestication history [22], all of which can lead to dramatic changes in microbiome properties. As the field moves towards better understanding of microbiome evolution in natural or semi-natural systems, we can now aim to: (i) predict and harness the microbiome’s evolutionary and functional responses to possibly enrich for particular microbiome traits; and (ii) understand and mitigate the impacts of potentially disruptive management methods of microbiomes and their effects on host fitness, which all have potential consequences for human health and agriculture.

In this review, we set out a framework for thinking about and differentiating between microbial evolution and ecology within communities, both in terms of methods used to detect these patterns and interpretation of results, all within the broader context of evolutionary theory. We first review the key findings and limitations from more simplified investigations of bacterial evolution in monoculture or two-species systems, and then discuss the distinction between ecological and evolutionary processes that lead to genetic changes in complex microbial communities highlighting recent experimental evidence of evolution in microbiomes. Recently, time-resolved metagenomic sequencing and experimentation have revealed that genetic changes can emerge and segregate among members of complex microbial communities such as the mice and human gut [11, 23, 24]. We explore how this and other new approaches would greatly increase our power to detect bacterial evolution in these ecologically complex settings and will take us one step closer to harnessing and manipulating microbiome function.

## Bacterial evolution in tightly controlled systems

**Figure 1:**
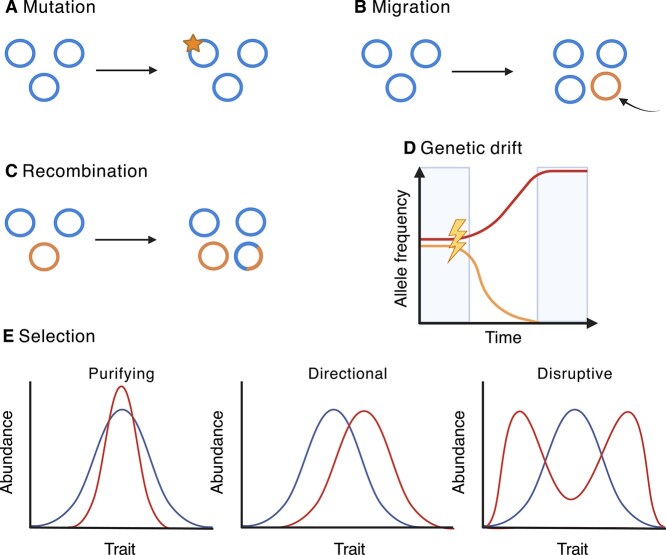
Visualizing the main evolutionary processes that lead to genetic change within populations of haploid bacteria chromosomes. (A–C) Bacterial chromosomes are simplified and represented by closed circles in which the same color refers to identical sequences across different chromosomes. (A) Mutation is perhaps the most well-known process leading to genetic change in bacteria, such that genetic changes, like single nucleotide polymorphisms or short insertions and deletions (depicted by the star), can arise de novo in a bacterial population and be maintained. (B) Migration is another source of genetic diversity such that if an individual migrates and establishes in the population, this can introduce genetic variability into the population. (C) Recombination allows for new genetic sequences and novel combinations of mutations/alleles to arise within a species. Often, homologous sequences are the basis for recombination allowing for entire segments of DNA to be shared, additionally, mobile genetic elements can also be responsible for this process though bacteriophage or plasmids. (D) Genetic drift is a change in frequency of a particular variant due to random chance, often this could be due to environmental catastrophes or bottlenecks. Drift can allow for rarer variants in a population to be maintained or even reach high frequencies if less rare variants are randomly lost. (E) Selection is a process underlying genotypic frequency changes on the basis of differential survival and reproduction. Here, initial populations are depicted with blue lines and final populations are red. With selection, individuals with more fit genotypes are more likely to survive and will increase in the population. Depending on the trait in question, selection can have significant impacts on the resulting trait variation. In purifying selection, variation is reduced towards the optimum. With direction selection the traits on one side the mean is preferred, such that average values and those on the other side become lower in frequency. Disruptive selection occurs when the average trait value is no longer the fittest and the two extremes increase in frequency.

Historically, bacteria have acted as key model organisms to study evolution as a process because of their rapid growth in highly controlled, laboratory settings [25]. These experiments leverage their large population sizes, short generation times, small genome sizes, and relative ease of laboratory use allowing for high replication [26]. The ability to freeze single clones or whole populations of bacteria throughout experimental evolution also allows for hypothesis testing and direct comparisons of ancestral and mutant/evolved genotypes, allowing for a “fossil record” of sorts. There are also many molecular and genetic techniques to both identify adaptive mutations and/or manipulate bacterial genomes to move from sequence to function. With such techniques, one can directly study how genetic change translates to important phenotypic change such as differences in gene expression, disease progression, or metabolic potential. Additionally, in the laboratory, ecological interactions can be easily controlled by starting experimental populations with a single clone/genotype and by using bacterial species that can be grown in vitro without an obligate host or an interacting partner (an obligate cross-feeder). Although bacterial evolution is often considered to be driven by mutation during asexual reproduction, the evolutionary forces that generate and maintain genetic variation in these taxa might be more similar to sexual organisms (Fig. 1), given the results of various experiments detailed in this review, and these forces are key to both microbiome function and maintaining species boundaries [27–29].

Among the most well-studied bacteria in evolutionary biology is the model organism *Escherichia coli* which has been leveraged to study the roles of selection, chance, and history on adaptation and evolution through long term evolution experiments in which replicate populations of a bacteria are maintained and serially passaged over time. The Long-Term Evolution Experiment (LTEE), started by Richard E. Lenski in 1988, is the longest running evolution experiment and has been pivotal towards understanding evolutionary dynamics [29]. The LTEE consists of 12 replicate populations of *E. coli* strain B that have undergone daily serial passaging in minimal media with limited glucose since its inception, allowing for over 75 000 generations of evolution to be tracked [30]. The grand-mean fitness trajectory across the entire experiment has been tracked, highlighting that the populations grow 70% faster than their ancestor in the LTEE environment, but that most of the fitness improvements occurred in the first few thousand generations of evolution [31]. The fitness trajectory fits a power-law model with no upper bound, suggesting that the fitness of these bacteria could increase indefinitely (the rate of improvement scales with the logarithm of time) as the experiment progresses [30, 31]. Whole genome sequencing of evolved clones sampled at various generations have been analyzed to examine the tempo and mode of genomic evolution, revealing a near-linear trajectory of genomic change over time in contrast to the fitness trajectory which demonstrated nonlinear adaptive change [32]. Additionally, adaptive changes within the experiment have been demonstrated through both parallelism of mutations at the genic level across populations as well as a higher rate of accumulation of nonsynonymous mutations compared to synonymous mutations over 50 000 generations [14].

The results of the LTEE shed light on the dynamics of evolutionary change over time in a constant environment. Subsequent monoculture evolution experiments have expanded this approach to test the effects of temperature stress on adaptive diversity at the molecular level [16, 33], strain specific evolution to minimal media [34], and the effects of historical contingency on adaptive potential [35, 36]. Like the LTEE, parallel mutations at the genic level are often identified across lines, but under these more stressful conditions beneficial mutations often occur in genes with large roles in the cell, such as the genes that encode RNA polymerase [34, 37]. Often, these first step mutations significantly alter gene expression, conferring a fitness advantage that allows the mutation to fix rapidly in the population [38]. Over time, especially if the experimental environment is constant rather than fluctuating, mutations that further amend gene expression and increase bacterial fitness (known as compensatory mutations), but with smaller effect can accumulate [39]. Despite this common result in vitro, natural populations tend to have highly conserved RNA polymerase sequences, suggesting that the molecular targets of evolution experiments in the lab may not be common mutational targets in complex environments, like a microbiome [37].

Even with their relatively simple design, monoculture experiments highlight the potential for rapid diversification within bacterial populations. For example, within the LTEE, researchers observed the emergence of an evolutionary innovation in a subpopulation that evolved new metabolic functions to utilize the carbon source citrate in oxic conditions after 31 000 generations of evolution [40]. Another such example in the LTEE resulted in cross-feeding interactions that evolved from a single ancestor resulting in two lineages called the L and S lineages. The L lineage utilized glucose more effectively than the S Lineage but secreted by-products that the S lineage could exploit. Genome sequencing coupled with expression analysis identified a minimal set of three mutations that allowed the S bacterial lineage to invade and stably coexist with the other [41]. Similar results exist from shorter experiments across other bacterial systems. *Pseudomonas fluorescens* was found to rapidly evolve in novel environments, displaying phenotypic differences in colony morphology that each have differences in niche specificity in a heterogenous environment [42]. The ability of an ancestral *P. fluorescens* to invade communities composed of the different niche specialist morphs was investigated. The study revealed that the ability of the ancestral *P. fluorescens* to diversify decreased with increasing niche occupation of a resident morph [43]. This suggests a resident niche occupant can inhibit the diversification of an invader, further underscoring how community dynamics (just like sub-population dynamics) are likely to look very different than clonal dynamics [43].

## Bacterial evolution within more ecologically complex systems

Although single species experiments have offered critical insight, experiments with increasing complexity are now uncovering significant and exciting dynamics between ecological interactions and bacterial evolution beyond what can be observed in simpler environments [44–46]. To address the consequences of rapid evolution on positive pairwise interactions, an elegant experiment was conducted with a two-species microbial system existing in either a constant or fluctuating environment [44]. By experimentally evolving *Acinetobacter johnsonii* and *Pseudomonas putida,* which are known to have differential competitive or cross-feeding interactions depending on the nutrient availability in the environment [47], the authors found evidence of parallel genetic changes across treatments. They conclude that rapid evolution in one species (even a single mutation) could destabilize positive species interactions and lead to extinction of the other highlighting the consequences of evolutionary change on community structure. An early experiment using a complex community of microbes experimentally evolved five species of bacteria from the *Fagus sylvatica* tree to investigate whether adaptation to new environments depends on the biotic environment [46]. The study revealed distinct evolutionary outcomes between species evolved in monoculture versus in polyculture. Reassembled communities built from polyculture-evolved isolates were more productive than communities reassembled from monoculture evolved isolates [46].

Indirect impacts of community members can also be examined with experimentation. In one study, the indirect effects of community complexity were studied by tracking 22 focal strains growing in physical separation (by dialysis bags) from bacterial communities isolated from rainwater [48]. The dialysis bags allowed for chemical exchange such that ecological interactions like resource competition and cell–cell signaling could still occur but direct contact and competition for space was blocked. Their investigation revealed that the capacity to evolve depended on both the identity of the focal strain (including its genome size), as well as the biodiversity of the background community. The primary genetic targets of evolution were those involved in carbon metabolism and cell–cell interactions, suggesting that substrate degradation and community interactions influence evolutionary trajectories in microbial communities and could select for novel community functions. In a plant-associated system, the effects of within-host selection on interactions between resident and invading species were investigated by passaging bacteria on their native plant hosts [49]. The focal bacterium, *Pantoea dispersa*, evolved in response to the tomato seedling environment in which it was passaged for 6 weeks and, in doing so, altered its competitive ability against a related species, *P. protegens*, and the plant pathogen, *Pseudomonas syringae*. The results again highlight how the ecological context in which evolution occurs can rapidly reshape microbiome community composition and relative abundance.

In a complex community of genomes, like a microbiome, we might usefully ask: when do we satisfy the criteria for genetic change through evolutionary processes versus ecological processes? Microbial communities are dynamic which makes it difficult to accurately measure intraspecies evolutionary dynamics and determine the contribution of emergence of de novo mutations versus invasion of new strains due to dispersal (e.g. through inter-host transmission). Although experiments like those described above are invaluable towards understanding evolution, often only mutational change in the form of de novo single nucleotide polymorphisms (SNPs), small insertions or deletions, or large structural variants are investigated and other important factors that may influence evolutionary change could be missed. For example, horizontal gene transfer (HGT) by way of recombination or other methods, like plasmid exchange, are important sources of genetic change that can be overlooked in many experimental designs.

Computational analyses of genome sequences from various bacterial species have revealed heterogeneity in the rates of recombination among different species of bacteria with different life histories [29, 50]. A recent study of sequenced fecal samples from humans have also shed light on the pervasiveness, variation, and contribution of recombination to the gut microbiome and its adaptability highlighting the need to understand it further [51]. To investigate such recombination experimentally, a modification to the classic Lenski experiment was conducted to also include the addition of four different donor *E. coli* strains during passaging every 5 days to investigate the effects of recombination through conjugation on the evolution of *E. coli* [52]. By sequencing the genomes of the evolved lines, the authors found that the effects of recombination were highly variable, such that each population varied in the amount of acquired donor DNA. The evolution experiment results highlighted that recombination could overwhelm selection, as donor alleles frequently reached fixation without a selective advantage in some cases.

## Designing and interpreting experiments to disentangle microbial ecology and evolution

**Figure 2:**
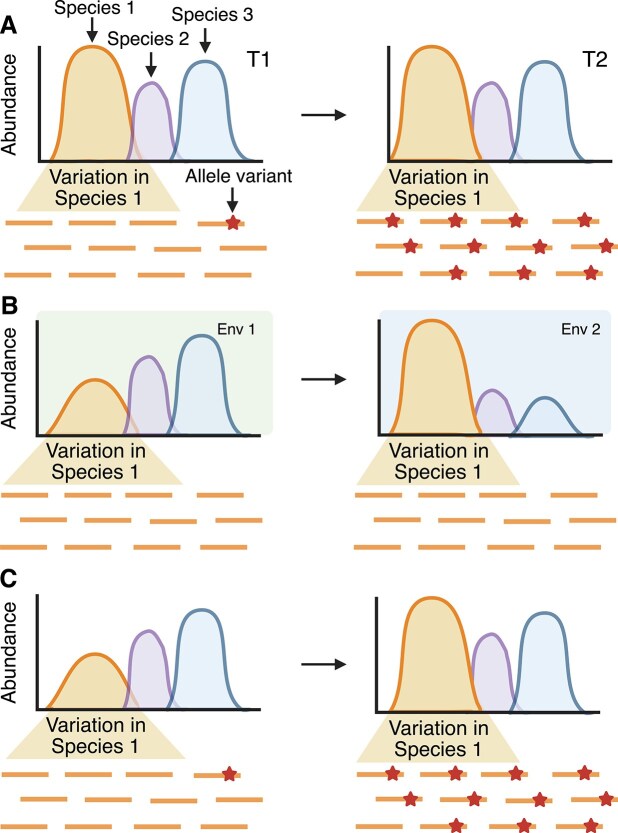
Changes in relative abundance of a simple microbiome under different scenarios to highlight the importance of genetic resolution and the contributions of ecology and evolution. In each panel, a mock microbiome community composed of three species is depicted over two time points (T1 and T2). Below each graph are cartoon sequencing reads corresponding to Species 1. (A) A toy example in which evolutionary change has occurred in Species 1 such that a de novo mutation has rose to high frequency, but it did not have an effect on the overall relative abundance of the community. (B) In this example, the environmental context differs such that Environment 1 (Env 1 in green) and Environment 2 (Env 2 in blue) have different abiotic characteristics resulting in different abundances of the three species. We must be cautious not to overstate changes in abundances measured by a particular gene marker (like 16S rRNA genes) as evolutionary change. Here, changes in microbiome composition as a result of selection from the abiotic environment or even simply neutral dispersal could occur only as a result of species turnover without any genetic changes within species. (C) Final example depicting evolutionary change influencing community structure, such that a de novo mutation emerges in Species 1, rises to high frequency, and affects its own relative abundance in the community. Ultimately, evolution shapes ecology such that species interactions can change as a result of evolutionary change. Here, a de novo mutation in one species led to phenotypic changes that affected its abundance in the community, as well as its competitive ability with other community members and/or its utility as a partner in terms of cross-feeding or physical structuring.

Studying evolutionary processes requires strain level resolution of bacteria, which can be difficult to achieve and often overlooked in microbiome experiments that use 16S rRNA gene sequencing to describe the community composition based on clustering of operational taxonomic units or quantifying amplicon sequence variants (ASVs). Such studies are cost effective and highly useful in determining the impact of certain factors, such as dietary intervention, nutrient availability, host filtering, or disease on microbiome community structure [53–55] and for applying ecological models of community assembly. As such, whole genome sequencing of isolated bacteria or metagenomic sequencing is necessary to study evolutionary processes and accurately define the mechanisms underlying changes in community abundances. Indeed, evolutionary changes or processes can be missed or completely masked when only the relative abundances of particular taxa are measured, and strain diversity is ignored **(**Fig. 2).

A bacterial strain is a particular subset of a bacterial species defined by genetic similarity, historically using a set of housekeeping genes, but which can also be determined with whole genome sequences [56, 57]. Over the last decade, whole genome sequencing has uncovered the extensive mosaicism found in gene content within a single species of bacteria [58]. In host-associated systems, this diversification can be so intense that it can result in vast genome reductions and cross-feeding among strains of a single species, as seen in the endosymbiont *Candidatus* Hodgkinia associated with cicadas [59]. Given that strains of a single species can carry entirely different sets of genes, often referred to as accessory genes, there is likely high heterogeneity in functional capabilities that are entirely masked by the use of marker genes. For studies of microbiome dynamics that rely on marker genes, the most common analyses rely on comparing relative abundance of different ASVs (representing strains or species depending on the resolution) across samples. This can be very useful in determining how two microbiomes differ (e.g. over space or time) but must be interpreted with caution if the question of interest relates to the fitness or impact of particular bacterial taxa [8]. This is because the observed relative abundance of one species is necessarily affected by changes in the abundance of the other species within the community. In cases where one previously dominant species decreases in absolute abundance, it might lead to what appears as an increase in the relative abundance of a second species even when the absolute abundance of the second species has not changed. This problem is further compounded because genetic changes like de novo mutations rising to fixation in one species would be missed if they are not accompanied by any changes in relative abundances observed at the ASV level (Fig. 2A). Similarly, a new strain might arrive in a population and replace an existing strain with identical 16S rRNA gene sequence, giving the impression of stability when evolution is rapidly occurring. Overall, changes in relative abundance of taxa over space or time can be indicative of responses to selection from the biotic or abiotic environment, but could also be the result of neutral dispersal or species turnover without any genetic changes within species (Fig. 2B), and it is critical to remember that these evolutionary and ecological processes can and do shape (feedback on) one another [60] (Fig. 2C).

To study ecology and evolution in concert, experiments could take inspiration from monoculture experiments, such as the LTEE, or take reductionist approaches like building synthetic microbial communities (SynCom). For example, to study the effects of biotic interactions on evolution one could evolve a focal bacterium with or without ecological interactions [45, 61] or one could passage whole communities across different selection pressures to elucidate the importance of evolutionary change versus ecological species sorting in microbiome dynamics [62]. With a well-built SynCom [63] (especially those where whole genomes for each member exist), one can study microbiome evolution with reduced complexity but still maintaining fundamental interactions that cannot be achieved with a single strain. Here, the evolve and resequence approach could be done at the metagenomic level, allowing for comparison of not just change in allele frequencies, but also movement of genes among taxa. Additionally, molecular tools such as genome wide transposon libraries have recently been utilized to study the effects of gene mutations during host colonization and can be leveraged across hosts and environments to study evolution in microbiomes [64]. In this case, barcodes associated with each starting lineage can allow for tracking of responses to selection (e.g. using Barcoded TnSeq approaches) or even neutral evolutionary change of just the focal strain in the presence of a complex microbiome.

## Importance of sequencing resolution in disentangling mechanisms of change

The term metagenomics was first introduced in 1998 by Handelsman et al. [65] and in its current context can be described as the totality of the genomic material present in a heterogenous microbial community [66]. With sufficiently deep sequencing and/or long read sequencing technologies, metagenome assembled genomes (MAGs) can also be recovered, allowing for analysis of the full set of genes found within a single bacteria, and thus the recovery of strain diversity from natural communities [67]. The assembly of MAGs is needed to describe the genetic variation that exists within bacterial populations which encodes its metabolic potential, though this task is not without its own difficulties [68–70]. Current difficulties include the inability to reliably close genomes, contamination from closely related genomes, and the computational resources needed to perform metagenomic assembly. Increasing sequencing depth or using long read sequencing technologies can aid in particular steps of the assembly process, like binning, to improve the accuracy of MAGs [71]. Although some seemingly pervasive and strong patterns of evolution like clonal interference (see **Focus Topic 1**) may suggest that multiple lineages may not exist simultaneously in a complex environment, other results from community settings or host-associated settings support the coexistence of multiple lineages within a single species as well as signatures of clonal interference by soft sweeps [41, 72]. Additionally, community function or productivity may be a product of multiple strains working together rather than just a singular genotype being sufficient [73, 74]. With metagenomic data, clearer definitions of bacterial species boundaries and total strain diversity within microbiomes in particular habitats could be inferred.

When interrogating a microbiome in any instance, experimental design is paramount in this case and has been extensively reviewed [75]. When studying evolution, one must be aware of the tradeoffs that exist when deciding community sequencing depth, coverage, and replication. As described above, marker gene analyses are cost effective and can work well in low-biomass samples, however, depending on the system, evolutionary change (genetic changes) could easily be missed. Metagenomic sampling typically calls for deeper sequencing depths which requires higher monetary costs and adequate computational resources, but it could allow for identification of genetic change by evolutionary processes (Fig. 1) [76] or ecological changes, such as species sorting [24].

Longitudinal sequencing, especially metagenomic sequencing, within the same host or environment provides valuable data to study evolutionary forces as genetic changes within species or populations can be tracked over time and the datasets can be used by the greater scientific community [24]. It is important to keep in mind that even though these datasets may be difficult to obtain, they can be an invaluable resource for the community, especially if researchers follow the FAIR data principles [77, 78]. Metagenomic datasets can also be reanalyzed and mined to study strain or ecotype diversity of bacteria [79–81].

Metagenomics skips biases arising from relying on culturing techniques and allows for profiling other microbes such as unicellular eukaryotes or bacteriophages that are also important components of the microbiome and themselves are important ecological factors that shape bacterial evolution. With sufficient depth, low abundance or rare organisms would also be identified that are likely to be missed with low sequencing depths. Finally, more theoretical approaches such as ancestral gene content reconstruction or machine learning techniques can provide valuable complementary insights and help predict evolutionary trajectories in microbial communities [82]. Incorporating such approaches can help generate testable hypotheses, improving our ability to design experiments that capture both ecological and evolutionary dynamics [83]. Overall, the field will better understand the standing genetic diversity that exists in natural environments, allowing for better predictions about evolvability and response to selection, and a better understanding of the species dynamics and boundaries in microbiomes [27].

## Current evidence of evolutionary change within microbiomes

**Figure 3:**
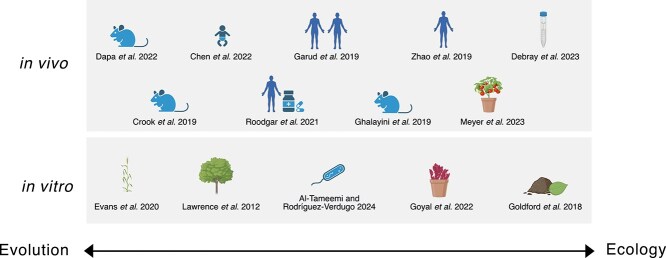
Ecology and evolution influence one another and occur on similar timescales. Depending on the study design, data collected, and analysis methods focus can be placed on one of these processes or on both of these processes at the same time. In general, experiments that study a single genetic locus can only explore ecological questions reliably, whereas experiments that utilized isolate or metagenomic sequencing are able to explore evolutionary questions. This figure highlights various microbiome studies described in the main text along a spectrum of evolution (left) and ecology (right) and between in vitro and *in vivo* experiments.

Although bacterial evolution can occur within hours and days, as evidenced by the experiments presented previously, there are lines of thought suggesting that evolutionary timescales are far too long compared to ecological change to have any significant effect in natural microbiomes [84]. For example, human associated microbes are known to have co-evolved with their hosts for millions of years [85], which may suggest that beneficial mutations should have already had an opportunity to fix in this time. However, time-resolved sequencing has illustrated that genetic changes can and do emerge and segregate within complex communities inside mouse and human guts within even a single host generation [11, 23, 24, 86–88]. This raises the intriguing possibility that within-host evolution can occur rapidly, and yet not have significant impacts on the diversity of the bacterial species (global population) as a whole due to different selection pressures resulting from transmission among hosts or survival in the wider environment. In disease ecology, the separation of within-host evolutionary effects and among-host selection has been particularly useful in explaining this apparent discrepancy [89]. Here, we review experimental and analytical studies of microbiomes or low-complexity microbial communities that illustrate evidence of genetic changes in bacteria through evolutionary processes (Fig. 3).

The mouse gut microbiome has provided an important system for studying bacterial evolution and demonstrated, e.g. how bacteria adapt to the gut environment through metabolism adjustments often involving gene inactivation [72, 90, 91]. Recently, strains of the model organism *E. coli* have been studied in their natural habitat, the mammalian gut, within a natural community context [10, 92]. By tracking the long-term evolution of maternally transmitted *E. coli* strain 536 in mice fed two different diets (a chow diet or a high-fat, high-sugar diet), analyses have demonstrated evolutionary change as a result of nonsynonymous point mutations, several short insertions or deletions, and structural variation in the form of two deletions >30 base pairs [10]. The distribution of the mutation types from the mouse gut generally mirrors the molecular changes previously identified in a laboratory strain of *E. coli* evolved to thermal stress in monoculture, laboratory conditions [16]. Additionally, evidence of selection was found during the breastfeeding period based on convergent evolution in the *lac* operon across diets, suggesting that mutations in the mother’s gut can be selected for during transmission to offspring. In other work, the evolution of *E. coli* strain Nissle, which is used as a probiotic, was studied across both germ-free mice and those harboring a SynCom composed of 13 human-gut derived bacteria [92]. The authors observed an *in vivo* mutation rate of 0.007 (+/− 0.002) SNPs per genome per generation and, in the mice colonized with the SynCom and the probiotic, there was evidence of functional convergence for carbohydrate metabolic processes. Research in the mouse gut on the evolution of *Bacteroides thetaiotaomicron,* which is the most abundant Gram-negative gut symbiont in humans [93], revealed unique evolutionary outcomes depending on the diet regimen (standard, Western, or alternating diet), such that intraspecies genetic diversity was lowest in mice kept on a constant diet and highest in those on an alternating diet [11].

Moving from experimental manipulation to an observational metagenomic approach, a longitudinal study of healthy human gut microbiomes tracked the evolution and diversification of *B. fragilis* [88]. Whole genome sequencing of over 600 isolates as well as stool metagenomes were used to identify adaptive mutations, as evidenced by parallel evolution in genes involved with polysaccharide utilization and cell-envelope biosynthesis. Similarly, the evolutionary dynamics of 40 prevalent gut bacteria species were investigated within and across human host gut metagenomes [87]. A model-based approach was utilized to quantify the evolutionary dynamics and identified evidence for the accumulation of genetic differences within hosts that were not attributable to ecological processes like strain replacements. In a study of mother and infant gut microbiomes over time, community changes were observed to be driven by both de novo mutations and transmission of pre-evolved lineages from family [24]. Transmission from mother to infant resulted in dynamic genomic changes like gene losses, highlighting mother-offspring transmission events as an important factor influencing evolution, echoing the *E. coli* strain 536 evolution in mice following mother-offspring transmission [10]. Within a single, human individual, the strain level changes in community composition before, during, and after antibiotic treatment were tracked in 36 species of bacteria [23]. The sequencing methods allowed for the identification of de novo change through SNPs. Within-species diversity amongst the 36 bacteria varied within the single individual, ranging from 100 to 10 000 SNPs. Their analysis of genetic changes suggests that the general pattern of species recovery following antibiotic treatment was driven by strain-level competition and evolution within the host rather than discrete recolonization events, highlighting the ability of evolution to act on similar timescales to ecological processes.

Just like those of gut-associated microbes, studies of microbiome evolution in plant communities have revealed important modes and consequences of microbiome evolutionary change that are moving us closer to improving agricultural health and maintaining ecosystem functioning. Using microbial communities from pitcher plants that were maintained in the laboratory, the co-evolution of closely related and distantly related community members in a complex environment were investigated [94]. Previously, laboratory evolution experiments have commonly found that a single SNP can destabilize ecological interactions [44], in the pitcher plant microbiome, however, the authors found that strains need a genetic distance of at least 100 base pairs to decouple ecological dynamics. Furthermore, the results suggested that strains are not ecologically equivalent: despite being from the same species, some strains could be more correlated with strains from another species than their own. Similarly to the focal *Bacteroides* evolution in the gut, *Stenotrophomonas* sp. Evolution was followed with or without a community of five co-occurring species on wheat straw [45]. Ultimately, evolutionary outcomes were dependent on the community presence and whether the community evolved with the focal bacteria or were kept static. Different metabolic functions were selected for based on the treatment, highlighting that the evolution and adaptation of new functions in bacteria may heavily rely on the species interactions and community dynamics.

Altogether, evolution experiments and longitudinal studies of microbiomes have come together to begin elucidating the patterns, tempo, and modes of evolution in these complex communities. Metagenomic and whole genome sequencing revealed that de novo mutations do occur in these complex environments, and these variants can even be transmitted from parent (host) to offspring (hosts) [24, 79]. Commonly, mutations in metabolic pathways were seen across experiments and environments [45, 88], which differs from the commonly seen mutations in global regulators identified in monoculture evolution experiments. Still, this remains an open, active area of research and more experimentation is needed across a range of hosts as well as investigations of all the members of the microbiome which also includes fungi, protists, archaea, and bacteriophage [95].

**Focus Topic 1: Understanding the effects of mutation at different resolutions**

**Figure 4:**
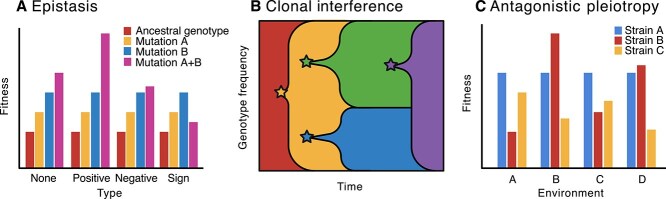
Impacts of evolutionary change in a community context from intraspecific to interspecific effects. Here, we summarize the effects of a mutation at different levels of resolution. (A) Different types of epistasis are depicted in the graph for an ancestral genotype and two beneficial mutations either alone or together on the ancestral genotype. Positive epistasis refers to a higher fitness effect of the two mutations together compared to the expected benefit. Negative epistasis refers to lower fitness in the double mutant genotype compared to the expectation when the two mutations are alone. Sign epistasis is a particular phenomenon in which the benefit of the double mutant is opposite to what is expected, e.g. if the double mutant had worse fitness than either mutation individually. (B) Clonal interference describes the phenomenon in which different adaptive mutations may arise and compete with one another in a single species. Here, different mutations are depicted by the different colors and arise directly from the genotype preceding it. (C) Antagonistic pleiotropy is a phenomenon that may underlie tradeoffs in evolution in which a mutation is beneficial in the selected environment but has deleterious effects in nonselected environments. Here, we depict three different strains of the same species of bacteria that have differential fitness in different environments. Strain A does not depict antagonistic pleiotropy, but Strain B does at different magnitudes.

Examining a mutation at different levels of resolution will assist in understanding the consequences that evolutionary change can have in bacterial communities. Imagine a simple genetic change in a bacterial chromosome, like a single nucleotide polymorphism (SNP) arising de novo within a gene sequence. Starting with a single species of bacteria at the genotypic level, genetic changes can have interactions with other portions of the same genome such that a mutation can have differing affects depending on the genetic background that it arises on (Fig. 4A). This phenomenon of genetic interactions among mutations that affect fitness is referred to as epistasis [96], and in sexually recombing organisms these interactions can be readily broken up and fine-tuned via meiotic recombination. Experiments that have quantified the contribution of epistasis to evolutionary change have illustrated the presence of epistatic fitness landscapes and differences in epistatic influence depending on the environment which can greatly influence microbial evolution [97–100]. Understanding the influence and extent of epistasis is imperative towards evolutionary predictions and can complicate the study of medically important topics such as antibiotic resistance if resistance is a result of gene-by-gene interactions.

If the de novo mutation described in the previous paragraph contributes some fitness benefit in this stable environment, it can begin a steady rise to fixation, replacing the ancestral genotype. A second, unrelated beneficial mutation could arise in the ancestral genotype that can then cause competition to occur between the different genotypes of this single species (Fig. 4B). The second mutation could outcompete the first adaptive mutation, eliminating an otherwise beneficial mutation from the population. This is known as clonal interference and is accepted as a prominent pattern seen in bacterial evolution, typically from monoculture evolution experiments [31]. Clonal interference may interact with other evolutionary forces in complex communities because of spatial structure, positive selection for strain diversity, and HGT [101].

A mutation may have different effects depending on the environment, including biotic and abiotic factors. In evolution, fitness tradeoffs are thought to be pervasive and can lead to differential phenotypic outcomes depending on environment. Antagonistic pleiotropy is the specific case in which a mutation is beneficial in the selected environment but has deleterious effects in nonselected (other) environments (Fig. 4C) [102, 103]. This phenomenon could have direct consequences on the evolution towards generalist versus specialist functions or lifestyles. Signatures of antagonistic pleiotropy have been found to influence *Pseudomonas* motility when colonizing *Arabidopsis thaliana* plants, suggesting the importance of the environment (context dependence) on mutation consequences [104].

**Focus Topic 2: Key ecological and evolutionary concepts with relevance to microbial communities**

*• Kill the Winner:* population dynamics property occurring during phage infection/predation such that fastest-growing organisms are more likely to be targeted, preventing those variants from sweeping the community subsequently maintaining rare variants in the population [105]. This concept does have a strong evolutionary parallel in the form of *Negative Frequency Dependent dynamics*, where parasites/predators are predicted to specifically adapt to, and decrease the evolutionary fitness of, common host/prey genotypes.

*• Black Queen Hypothesis*: provides a theory of reductive evolution explaining how selection leads to dependencies between organisms through gene losses [106]. This is similar to the concept of *Compensated Trait Loss* where interacting organisms lose or repurpose genes that provide functions redundant in light of ecological interactions [107]. This can lead to genome streamlining/reduction, but also fuel evolutionary innovation.

*• Priority effects*: results when the order of arrival for dispersing species to a unique local environment impacts the success of the arriving taxa and thus shapes community assembly [108]. These can result from facilitation, whereby subsequent species benefit from the earlier arrival of others, inhibition, where dispersing species are unable to establish in the presence of particular taxa that arrived previously, or pre-emption, where the niche is altered upon species arrival.

*• Monopolization*: a specific type of priority effect in which the first/earlier taxa to arrive to a unique local environment adapts through genetic changes that prevent subsequent invasion by later arriving taxa [109].

*• Evolutionary rescue*: a phenomenon by which populations experiencing severe or lethal stress may avoid extinction through adaptation by natural selection, facilitated by the emergence of a de novo mutation in a microbe, e.g. [110].

*• Eco-evolutionary feedback*: a process by which evolutionary change alters the ecological interaction among species, subsequently changing the selection pressures underlying the interaction, which then reshapes the future evolutionary trajectories of the populations, and ultimately their ecology.

*• Allee effects*: a density-dependent phenomenon in which the per capita growth rate is reduced at lower population sizes compared to larger populations [111]. Ecological factors such as predation and interactions by cross-feeding can underlie these dynamics.

## Conclusions

The existing data support that ecological and evolutionary processes occur on similar timescales in microbiomes and genetic changes through evolutionary processes do occur in natural microbiomes which can have significant effects on microbiome function and host health. Overall, there is a medical and environmental need to better understand microbiome function, and as we have highlighted throughout this review, such change is likely to represent tightly intertwined ecological, evolutionary, and eco-evolutionary processes. Designing experiments to explicitly test for and differentiate ecological and evolutionary processes in microbial communities and microbiomes across a variety of hosts and environments will be essential to fully leverage our knowledge to engineer or direct evolution of microbiomes to particular functions or states. Careful thought must be given to when and how such effects can be disentangled as, although acting at similar spatial and timescales, these processes are critically different in their impacts on microbial systems.

## Data Availability

This article is a review of existing literature. No new data were created or analyzed in this study.
